# 604 From admission to discharge: a total friction burn review from a single institution

**DOI:** 10.1093/jbcr/irac012.232

**Published:** 2022-03-23

**Authors:** Oscar I Estrada Munoz, Andrew F Sabour, Joseph T Carroll, Carmen E Flores, Douglas R Fraser, Paul J Chestovich, Syed F Saquib

**Affiliations:** Kirk Kerkorian School of Medicine at UNLV, Las Vegas, Nevada; Kirk Kerkorian School of Medicine at UNLV, Cupertino, California; University of Nevada Las Vegas, Las Vegas, Nevada; Kirk Kerkorian School of Medicine at UNLV, Las Vegas, Nevada; Kirk Kerkorian School of Medicine at UNLV, Las Vegas, Nevada; UNLV School of Medicine, Las Vegas, Nevada; Kirk Kerkorian School of Medicine at UNLV, Las Vegas, Nevada

## Abstract

**Introduction:**

Most friction burns are adequately managed in an outpatient setting. However, many require hospital admission, operative excision, and extended care especially those that present at trauma or burn centers. There is a wide variance in friction burn management. Our goal is to review the etiology, management, and outcomes of such burns warranting hospitalization.

**Methods:**

We conducted a retrospective review of all friction burns admitted to a single, American Burn Association verified burn center from January 1, 2016 to December 31, 2020. Individual chart analysis was performed using data from the hospital’s burn registry. Statistical analysis was performed using Chi-square and Wilcoxon rank-sum test with p< 0.05 being significant.

**Results:**

Eighty-two patients met the inclusion criteria. Mean age was 35.4 years (95% CI 31.6-39.2). The overall mean Total Body Surface Area (TBSA) was 9.0 % (95% CI 7.5-10.6), and mean TBSA of 3^rd^ degree burns was 1.1 % (95% CI 0.6-1.7). The most common mechanism of injury was motorcycle collision (45, 55%), followed by pedestrian struck by automobile (13, 16%). Fifty-four individuals (65%) had a concomitant injury. The most common topical agent used was silver sulfadiazine (52%), followed by bacitracin (21%). Sixteen patients (20%) required ICU level of care. Twenty-eight (34%) patients required surgery for their friction burns and 15 (18%) ultimately required a split-thickness skin graft. The mean number of operations was 2.4 (95% CI 1.6-3.1).

Overall, the operative group was younger (29.9 vs 38.3 years, p=0.026), more likely to have a concomitant traumatic brain injury (25% vs 7%, p=0.027) and had a longer hospital length of stay (17.5 vs 3.9 days, p< 0.001). Both groups had a similar overall TBSA (8.5% vs 10.0%, p=0.35), but the operative group had larger surface area comprised of 3rd degree burns (3.05% vs 0.2%, p< 0.001). Eighty-one patients survived with the sole death due to massive hemoptysis.

**Conclusions:**

Friction burns resulting in hospital admission are associated with high-energy traumatic mechanisms and concomitant injuries. Patients who need operative intervention of their burns typically require multiple procedures often culminating in a split-thickness skin graft.

